# Protective Effect of the α7 Nicotinic Receptor Agonist PNU-282987 on Dopaminergic Neurons Against 6-Hydroxydopamine, Regulating Anti-neuroinflammatory and the Immune Balance Pathways in Rat

**DOI:** 10.3389/fnagi.2020.606927

**Published:** 2021-01-25

**Authors:** Ying Jiang, Huizi Ma, Xuemei Wang, Zhan Wang, Yaqin Yang, Longling Li, Tao Feng

**Affiliations:** ^1^Center for Movement Disorders Disease, Department of Neurology, Beijing Tiantan Hospital, Capital Medical University, Beijing, China; ^2^Parkinson’s Disease Center, Beijing Institute for Brain Disorders, Beijing, China; ^3^China National Clinical Research Center for Neurological Diseases, Beijing, China; ^4^Department of Neurology, Zhongshan Hospital, Xiamen University, Xiamen, China

**Keywords:** α7 nicotinic acetylcholine receptors, PNU-282987, Treg, immune balance, Parkinson’s disease, rat, 6-hydroxydopamine

## Abstract

Neuroinflammation and inner immune dysfunction are increasingly accepted as important components of the etiopathogenesis of Parkinson’s disease (PD). According to emerging evidence, a7 nicotinic acetylcholine receptor (α7nAChR), a ligand-gated ion channel, plays an important role in inflammatory reactions and is also expressed on the surface of T cells. In particular, regulatory T cells (Tregs) are critical for the maintenance of immunological tolerance. In the present study, we investigated the roles of α7nAChR in inhibiting inflammation and maintaining the immune balance in rats with 6-hydroxydopamine (6-OHDA)-induced lesions and the possible mechanisms regulating the proportion of Tregs *in vivo*. Adult male Wistar rats (*n* = 90) were subjected to a unilateral injection of 6-OHDA into the left medial forebrain bundle, and PNU-282987, an α7nAChR agonist, was intraperitoneally injected 2 h prior to the induction of lesions by 6-OHDA and again at days 1, 7, and 13 postlesion. Behavioral tests and immunohistochemical staining to detect the expression of tyrosine hydroxylase (TH) in the bilateral substantial nigra (SN) were performed. Subsequently, CD4+ T lymphocytes and the expression of forkhead/winged helix transcription factor p3 (Foxp3, which is a marker of Treg cells) in the SN were also assessed using immunofluorescence staining. The expression of glial fibrillary acidic protein (GFAP) in the SN was determined by performing immunohistochemical staining. Additionally, the protein levels of α7nAChR, extracellular signal-regulated kinase (Erk) phosphorylated-Erk (p-Erk) and Foxp3 in the ventral midbrain were determined using Western blotting, and the relative expression of the TNF-α, IL-1β, and IL-10 mRNAs were detected using real-time quantitative reverse transcription-polymerase chain reaction (RT-PCR). We found that PNU-282987 significantly improved the motor deficits induced by 6-OHDA, reduced the loss of TH in the SN, suppressed the overactivation of GFAP+ cells and expression of related inflammatory cytokines, and increased the number of Foxp3+ cells. In addition, we also showed that PNU-282987 significantly increased the protein expression of the a7nAchR, p-Erk, and Foxp3 in 6-OHDA-lesioned rats (*p* < 0.05). These results indicated that α7nAChR activation could exert an anti-inflammatory effect and participate in the process of modulating the immune balance during 6-OHDA-induced injury, potentially through the α7nAChR/p-Erk/Foxp3 signaling pathway.

## Introduction

Parkinson’s disease (PD), which is one of the most common neurodegenerative diseases in the aging population, is characterized by the progressive and selective destruction of dopaminergic neurons in the substantia nigra pars compacta (SNpc) in the brain (Tysnes and Storstein, 2017; de Campos et al., 2020; Jayaraj et al., 2020). Although the etiology of the disease remains unknown, accumulating evidence shows that neuroinflammation and mitochondrial dysfunction are common features of PD pathology, and recent studies have identified an important role for the adaptive immune system in the development of PD (Harms et al., 2017; Manocha et al., 2017; Park et al., 2017).

Clinical studies have revealed that an increase in the proportion of activated (CD4+ CD25+) helper T cells accompanied by the upregulation of Fas expression trigger susceptibility to apoptosis and nitrated-alpha-synuclein (Calopa et al., 2010). In a PD mouse model, Tregs inhibit excess immune responses to reduce the MPTP-induced dopaminergic neuron damage (Brochard et al., 2009). Together, these results suggest that immune dysfunction might be a potential mechanism in the PD model and that CD4+ T cells are closely associated with PD pathogenesis and progression.

It has also been reported that Th 17 cells and Tregs are both critical for the maintenance of immunologic homeostasis (Zhu et al., 2017). Th17 cells act as a pro-inflammatory factor and appear to be essential for the pathogenesis of many inflammatory diseases. Furthermore, these proinflammatory cytokines induce glial overactivation, which results in increased release of inflammatory cytokines and thereby a neuroinflammatory cascade and neurodegeneration (Gao et al., 2003; Zimmermann et al., 2013). In contrast, growing evidence suggests that Treg cells can potently inhibit the function of Th1, Th2, Th17, and other effector cells and preserve autoimmunity (Josefowicz et al., 2012; Tao et al., 2017). Based on accumulating evidence, Treg cells are a critical regulator of immunosuppression and immune system homeostasis (Shuping et al., 2020). TGF-β1 and IL-10 secreted by Tregs also exert immunosuppressive effects, and the upregulation of forkhead/winged helix transcription factor p3 (Foxp3) expression efficiently induces the differentiation of T cells into the Treg phenotype (Deng et al., 2019). Currently, novel clues suggest that Treg cells might exert protective effects on inhibiting PD-related neuroinflammation and immune activation (ReynoLSD et al., 2007).

As previously described, a7 nicotinic acetylcholine receptor (α7nAChR), a ligand-gated ion channel, is widely expressed in neurons, endothelial cells, microglial cells, monocytes, macrophages, dendritic cells, neutrophils, and T and B lymphocytes (Wang et al., 2004). Notably, α7nAChR plays an important role in suppressing the synthesis of proinflammatory cytokines in macrophages and glial cells (Wang et al., 2003; Parada et al., 2013). As shown in the study byShytle et al. (2004), the activation of α7nAChR expressed on microglia inhibits LPS-induced TNF-α production. Moreover, α7nAChR also plays an important role in controlling the apoptosis of T cells and the development and antibody secretion of B cells, indicating that the receptor is a critical regulator of immune function (Wang et al., 2004). Recently, mounting evidence shows that α7nAChR activation is neuroprotective against toxin-induced dopaminergic cell loss in the SN of a PD animal model (Suzuki et al., 2013). In our previous study, we found that aVNS appears to maintain the immune balance and promotes neuronal integrity in a PD animal model, and this effect might occur through α7nAChR activation (Jiang et al., 2018). The protective effect of α7nAChR has been linked to inflammation and the regulation of the immune balance. However, little information is available on the mechanisms by which α7nAChR contributes to the immune balance in PD (Cui and Li, 2010; Liu et al., 2015; Kalkman and Feuerbach, 2016).

We conducted this study to determine whether the activation of α7nAChR could exert neuroprotection against 6-OHDA-induced lesions and modulate the inflammation and inner immune balance related to PD, particularly from the perspective of Treg cells. Therefore, we performed the present study with the 6-OHDA-lesion group as the reference group. In addition, we further investigated the possible molecular mechanisms responsible for the α7nAChR-mediated regulation of CD4+ T cells *in vivo*.

## Materials and Methods

### Animals and Reagents

Adults male Wistar rats (*n* = 90) with a body weight of 200–250 g were purchased from Beijing Vital River Laboratory Animal Technology Co., Ltd., Beijing, China. The animals were maintained under standard laboratory conditions, which consisted of a controlled temperature of 24 ± 2°C with 60% humidity and a 12-h dark/12-h light cycle. Standard food and water were provided *ad libitum*. All experiments were performed according to the Regulations of Experimental Animal Administration issued by the State Committee of Science and Technology of China (promulgated by the Decree No. 676 of the State Committee of Science and Technology of China on March 1, 2017). The neurotoxin 6-OHDA and the α7nAChR agonist (PNU-282987, N-[(3R)-1-azabicyclo[2,2,2]oct-3-yl]-4-chlorobenzamide hydrochloride) were purchased from Sigma-Aldrich (St. Louis, MO, United States).

### Experimental Design

**FIGURE 1:**
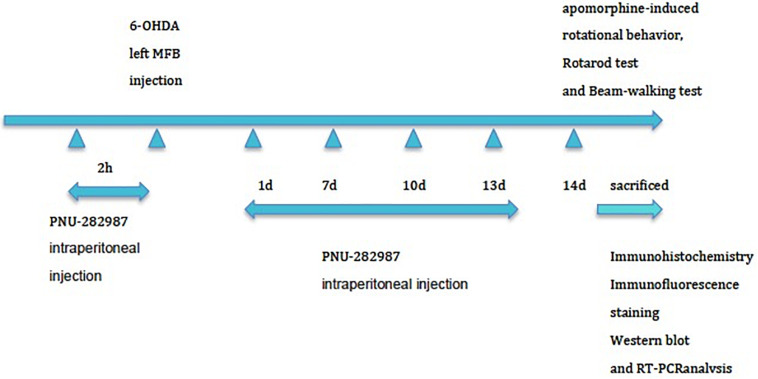
Experimental protocol. The three groups (*n* = 90) were subjected to stereotaxic surgery: group 1: 6-OHDA-lesion group, group 2: 6-OHDA-lesion + sham group, and group 3: 6-OHDA-lesion + PNU-282987 (α7nAChR agonist) group. For the PNU-282987 treatment, rats received intraperitoneal injections of PNU-282987 at a concentration of 3 mg/kg 2 h prior to lesion induction with 6-OHDA and then at days 1, 7, and 13 postlesion. Rats in the 6-OHDA-lesion + sham group received intraperitoneal injections of the equivalent amount of saline at the same time points. The rats (*n* = 6 per group) were sacrificed on day 14 for TH staining after assessing the motor deficits. Then the other rats were also sacrificed for morphological (*n* = 12 per group), Western blot (*n* = 6 per group), and RT-PCR (*n* = 6 per group) analysis at 14 days postlesion.

The experimental animals (*n* = 90) were randomly assigned to one of three groups: 6-OHDA-lesion group (*n* = 30), 6-OHDA-lesion + sham group (*n* = 30), and 6-OHDA-lesion + PNU-282987 (α7nAChR agonist) group (*n* = 30). A total of 30 rats were included in each group. In each group, 6 rats were for the behavior tests, and then for the morphological analysis together with another 12 rats, another 6 rats were for the western blot, and the remaining 6 rats were the for RT-PCR analysis. Briefly, the animals (280–340 g) were deeply anesthetized with isoflurane (4%, 500 mL/min) and were maintained under anesthesia with 2.5% isoflurane (500 mL/min) during surgery. The MFB lesion protocol with 6-OHDA rapidly produces a substantial dopaminergic lesion (Truong et al., 2006). Animals were fixed to the stereotaxic frame (David Kopf Instruments, Tujunga, CA, United States) and injected with the neurotoxin 6-OHDA (Sigma-Aldrich, St. Louis, MO, United States) into the left MFB as previously described to establish the rat PD model (Blesa et al., 2012). Two microliters of a solution containing 8 μg of 6-OHDA [dissolved in 0.9% NaCl containing 0.1% ascorbic acid (Sigma Aldrich, Poland)] were infused (delivered over 2 min) using a Hamilton syringe (25-gauge, Hamilton, Massy, France) at the following coordinates relative to the bregma: bregma −4.0 mm, lateral −0.8 mm, and ventral −8.0 mm). The needle was left in place for an additional 5 min at the end of the injection and then slowly withdrawn at a rate of 1 mm/min. As shown in Figure 1, for the PNU-282987 treatment, six rats received intraperitoneal injections of PNU-282987 at a concentration of 3 mg/kg 2 h prior to lesion induction with 6-OHDA and then at days 1, 7, and 13 postlesion in order to induce a potential neuroprotective effect during the experiment by repeatedly activating α7AChR. Rats in the 6-OHDA-lesion + sham group received intraperitoneal injections of the equivalent amount of saline at the same time points. On day 14 postlesion, rats (*n* = 6 per group) underwent behavioral testing and then were sacrificed for morphological, Western blot, and RT-PCR analysis.

### Behavioral Testing

#### Apomorphine-Induced Rotational Behavior

As described previously, the number of rotations of an animal treated with apomorphine is widely used to evaluate the extent of dopamine depletion following the induction of unilateral 6-OHDA lesions in rat models of PD (Wang et al., 2013). On day 14 postsurgery, apomorphine-induced rotations were observed for 60 min after a subcutaneous injection of 0.5 mg/kg apomorphine (dissolved in a 0.1% ascorbic acid solution) in an automated rotameter bowls, with tethers attached to the skin of the nuchal region (Chen et al., 2018; Rosa et al., 2020). Then, full rotations were recorded. The net number of rotations was expressed as contralateral minus ipsilateral full turns (*n* = 6 per group).

### Rotarod Test

This test is widely used for evaluating motor coordination, balance and ataxia (Khuwaja et al., 2011; Dräger et al., 2012). The apparatus used in this experiment consisted of a rotating rod with a 60-mm diameter, and the test was started with a rotation speed of 5 cycles per min with a cut-off time of 5 min. Before the test, each rat was allowed to habituate to the rotarod apparatus for three consecutive days. The results were presented as the mean latency to fall from the rod in three consecutive trials, and a 5-min rest period was included between each set of trials (*n* = 6 per group).

### Beam-Walking Test

The motor performance and coordination of the rats were evaluated using the beam-walking test. The testing apparatus consisted of a round wooden beam with a length of 110 cm, a width of 9 cm, and a thickness of 3 cm and fixed 7.5 cm above a countertop with additional supports. The animals (*n* = 6 per group) were trained to traverse the beam once for three consecutive days before the test. In the tests, the traveling time in three trials was recorded.

### Immunohistochemistry

The rats (*n* = 6 per group) were deeply anesthetized with a mixture of ketamine (60 mg/kg) and xylazine (10 mg/kg), and then sequentially transcardially perfused with 0.9% saline and cold 4% paraformaldehyde (Sigma-Aldrich, Inc., St. Louis, MO, United States) dissolved in 0.01 M PBS at pH 7.4. Briefly, the brains were removed, postfixed overnight with 4% paraformaldehyde at 4°C, cryopreserved through a 10–30% sucrose gradient in 0.1 M PBS at 4°C, and embedded in paraffin. Coronal sections (with a thickness of 5 μm) through the SN plane were cut using a microtome (Finesse 325, Beijing Sinopatho Technology Co., Ltd., China). One series from every sixth section containing SNpc (a total of 19–24 sections per rat) were selected. The prepared sections were then dewaxed in xylene, passed through a decreasing alcohol gradient, and subjected to antigen retrieval in 0.01 M citrate buffer (pH 6.0; Sigma). The sections were incubated with 3% H_2_O_2_ (Ainuo Chemical Products Limited Company, Tianjin, China) for 10 min and blocked with 10% normal goat serum (Cell Signaling Technology, MA, United States) for 20 min at 4°C. Free-floating sections were incubated overnight with a tyrosine hydroxylase (TH) antibody (1:1000, Abcam) or glial fibrillary acidic protein (GFAP) antibody (1:50, Cell Signaling Technology) at 4°C. Thereafter, the sections were rinsed with 0.1 M PBS, incubated with the secondary antibody (Dingguo Changsheng Biotechnology Co., Ltd., Beijing, China) for 40 min. Finally, the sections were stained with diaminobenzidine (DAB), then counterstained with hematoxylin (Dingguo Changsheng Biotechnology Co., Ltd., Beijing, China) staining for 5 min, followed by hydrochloric acid alcohol differentiation and immersed in ammonia solution, dehydrated in a graded series of alcohol solutions, cleared with xylene and coverslipped. As described in our previous studies (Jiang et al., 2018), unbiased stereological methods were used to analyze cell counts analysis was performed. After the identification of the regions of SNpc boundaries under a low magnification objective (4×), the stereological analysis was performed at 100× magnification with Stereo Investigator software (MBF Bioscience Inc., Williston, VT, United States). The stereological parameters (counting frame and sampling grid) were calculated as described in a previous study (Hudson et al., 1993; Andrzejewski et al., 2017). The optical fractionator stereological probe was used for cell counts and only cells coming into focus through the sampling brick were counted. The results were expressed as the percentage of TH+ cells on the lesioned SN with respect to the contralateral (unlesioned) SN. The number of GFAP-positive cells and the total cells on the lesioned side (counterstained with hematoxylin) were counted under a light microscope (CX21; Olympus Corporation, Tokyo, Japan) at 200× magnification by the same independent investigators. The average number of GFAP-positive cells and total cells were selected randomly from five visual fields and analyzed using Image-Pro Plus 6.0 software (Media Cybernetics, Inc., MD, United States). The result was reported as the percentage of GFAP-positive cells on the lesioned side with respect to total cells on the ipsilateral side.

### Immunofluorescence Staining

The rats (*n* = 6 per group) were anesthetized with a mixture of ketamine (60 mg/kg) and xylazine (10 mg/kg), and transcardially perfused as described above. The rat brains were rapidly removed, postfixed overnight in PFA and then transferred to 30% sucrose in 0.1 mol/L phosphate buffer for cryoprotection. According to the brain atlas of Paxinos and Watson (1997), serial, coronal, 10-μm-thick frozen brain sections through the SN (antero-posterior, bregma: −4.8 mm) were sliced on a cryostat (Thermo Fisher Scientific, MA, United States) at −15°C. Five sections per rat at the coronal level −5.4 mm from bregma were selected. The sections were thawed at 25°C for 30 min and preincubated with 0.4% Triton X-100 for 10 min, blocked with 10% normal donkey serum for 90 min, and then incubated overnight at 4°C with the following primary antibodies: CD4 antibody (1:100, AbD Serotec) or Foxp3 antibody (1:100, Novus). The sections were then sequentially incubated with secondary antibodies (anti-mouse IgG Fab2 Alexa Fluor 594; 1:200 and Anti-rabbit IgG Fab2 Alexa Fluor 488;1:200, CST, MA, United States) for 1 h at room temperature. Next, the slices were washed 3 times with PBS, counterstained with DAPI (Vector Laboratories, United Kingdom) for 10 min, and then washed again with PBS for 30 min. The slices were stored in the dark (−20°C). Finally, images were captured at 400× magnification using a laser-scanning confocal microscope (Leica TCS SP2, Wetzlar, Germany). The numbers of Foxp3 positive cells and CD4+ cells were blindly counted using the Image Tools software.

### Western Blot Analysis

The rats (*n* = 6 per group) were deeply anesthetized with a mixture of ketamine (60 mg/kg) and xylazine (10 mg/kg), decapitated. According to the brain atlas of Paxinos and Watson (1997), the ventral midbrain tissues (*n* = 6 per group), mainly SN (coordinates: antero-posterior, bregma: −4.8 mm; lateral: 1.8 mm; ventral dura: 9.2 mm) and Ventral Tegmental Area (coordinates: antero-posterior, bregma: −4.8 mm; lateral: 1.0 mm; ventral dura: 9.2 mm), were rapidly removed to the glassware in an ice box with the fine iris scissors. The rapid method for ventral midbrain dissection was performed as previous described (Heffner et al., 1980). The obtained brain tissues were homogenized in lysis buffer (Beyotime Inst. Biotech, Beijing, China) containing a cocktail of protease inhibitors for total protein extraction and assayed according to a previous report (Lee et al., 2020). Briefly, homogenates was centrifuged at 12,000 rpm for 30 min at 4°C. The protein concentrations were determined using a BCA Protein Kits (Pierce, Rockford, IL, United States). The protein levels were normalized and proteins (40 μg from each sample) was separated by 10% SDS-PAGE and then immunoblotted onto polyvinylidene difluoride membranes (Millipore, Billerica, MA, United States). After transfer, the membranes were blocked with 5% non-fat milk in TBS for 2 h and then incubated with rabbit anti-α7nAChR (1:1000, Abcam), rabbit anti-p-Erk, Erk (1:1000 Abclonal), and mouse anti-Foxp3 (1: 2000, Novus) primary antibodies or a mouse anti-β-actin (1:5000; Sigma) primary antibody overnight at 4°C. The blots were washed, incubated with HRP-conjugated secondary antibodies (goat anti-rabbit IgG;1:5000 and goat anti-mouse IgG; 1: 5000 Dingguo Changsheng Biotechnology Co., Ltd., Beijing, China) for 2 h at 37°C. The bands were visualized using an enhanced chemiluminescence reagent kit (GE Healthcare, Chalfont St. Giles, United Kingdom), detected with X-ray film and quantified using Quantity One software (Bio-Rad Laboratories, Inc., CA, United States). The optical density of each protein band was normalized to β-actin (Cell Signaling Technologies, Beverly, MA, United States) as an internal control.

### RNA Isolation and Real-Time Reverse Transcriptase (RT)-Polymerase Chain Reaction (PCR)

The rats (*n* = 6 per group) were deeply anesthetized with a mixture of ketamine (60 mg/kg) and xylazine (10 mg/kg) and decapitated. RNA was purified using the TRIzol reagent (Invitrogen Corp.) and the RNeasy Mini Kit (QIAGEN Sciences). The primers used for the analysis are shown in Table 1 (GenBank database, NCBI). Total RNA (*n* = 6 per group) was isolated from the ventral midbrain using the TRIzol reagent (Gibco BRL, Rockville, MD, United States) according to the manufacturer’s instructions. The RNA samples were resolved on an agarose gel to assess the integrity of the 18 S and 28 S rRNAs. A260/A280 ratios of the purified RNA samples ranging from 1.8 to 2.0 were determined using a UV-Vis spectrophotometer (Quawell Q5000, United States). The cDNA templates were synthesized from 2 μg of total RNA in a total reaction volume of 25 μL using a reverse transcription kit (Takara, Japan). The conditions for amplification were as follows: 95°C for 5 min and 40 cycles of 10 s at 95°C and 30 s at 60°C. Each amplification from the different RT reactions was repeated three times. Ct: the fractional cycle number at which the fluorescence reaches a certain threshold. ΔCt = Ct(target gene) – Ct(reference gene), the difference in Ct between a target and a reference gene within the same sample. ΔΔCt = ΔCt(target sample) – ΔCt(reference sample), the difference in ΔCt between the target and reference sample. The data are presented as 2^–ΔΔCt^, which is the amount of the target mRNA normalized to the endogenous reference and relative to a reference sample.

**TABLE 1:** Primer sequences.

**Gene**	**Sequences**
**TNF-α** (NM_012675)	F: 5′-CCACCACGCTCTTCTGTCT-3′
	R: 5′-GGCTACGGGCTTGTCACTC-3′
**IL-1β** (NM_031512.2)	F: 5′-TCTGTGACTCGTGGGATG-3′
	R: 5′-CTTGTTGGCTTATGTTCTGT -3′
**IL-10** (NM_012854)	F: 5′-AGTCAGCCAGACCCACAT-3′
	R: 5′-GGCAACCCAAGTAACCCT-3′
**GADPH** (NM_017008)	F: 5′-ATGATTCTACCCACGGCAAG-3′
	R: 5′-CTGGAAGATGGTGATGGGTT-3′

### Statistical Analysis

The data are presented as the means ± standard errors of the means (SEMs) and were analyzed using SPSS 17.0 software (SPSS Inc., Chicago, IL, United States). Student’s unpaired *t*-tests were used for comparisons between two groups, and the data from multiple groups were compared using ANOVA followed by the LSD test. A value of *p* < 0.05 was considered statistically significant.

## Results

### PNU-282987 Rescues the 6-OHDA-Induced Behavioral Deficits in Rats

**FIGURE 2:**
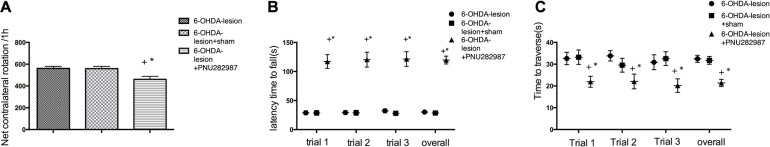
The PNU-282987 treatment partially improved the motor deficits induced by 6-OHDA. **(A)** Apomorphine-induced rotational behavior. **(B)** Mean latency to fall. **(C)** Time to reverse. The data are presented as the means ± SEMs (*n* = 6 per group). ^+^*p* < 0.05 compared with the 6-OHDA-lesion group, and *p < 0.05 compared with the 6-OHDA-lesion + sham group.

Apomorphine-induced contralateral rotations were assessed on day 14 to examine the effects of PNU-282987 on the modulation of dopaminergic transmission. As shown in Figure 2A, the lesioned rats showed a significant increase in the number of rotations (561.0 ± 18.61 contralateral rotations/h) after the apomorphine injection. PNU-282987 significantly reduced the number of contralateral rotations/h to 461.3 ± 24.73 compared with the 6-OHDA-lesion group (*p* = 0.005) and 6-OHDA-lesion + sham group (*p* = 0.006) (the data are listed in Supplementary Table 1). One-way ANOVA followed by the LSD as the *post hoc* test for multiple comparisons detected significant differences in the net number of contralateral rotations [*F*(2,15) = 7.04, *p* = 0.007].

The three groups of rats were subjected to rotarod and beam-walking tests on day 14 to assess whether the PNU-282987 treatment mitigated the 6-OHDA-induced locomotor behavioral deficits in rats. As shown in Figure 2B, based on the results of the one-way ANOVA with the LSD *post hoc* test, the 6-OHDA-lesion + PNU-282987 group showed significant improvements in motor deficits that were characterized by a markedly longer latency to fall in three trials separately [*F*_*trial*__1__/__2__/__3_(2,15) = 45.84/43.45/44.89, *p*_*trial*__1__/__2__/__3_ < 0.001] and [*F*_*overall*_ (2,51) = 150.91, *p* < 0.001] compared to the 6-OHDA-lesion and 6-OHDA-lesion + sham groups (the data are listed in Supplementary Table 2). In the beam walking test (Figure 2C), a decreased time to travel the beam was observed for the 6-OHDA-lesion + PNU-282987 group [*F*_*trial*__1__/__2__/__3_(2,15) = 4.704/3.904/4.355, *p*_*trial*__1__/__2__/__3_ = 0.026/0.043/0.032] and [*F*_*overall*_ (2,51) = 13.52, *p* < 0.001] compared to the 6-OHDA-lesion and 6-OHDA-lesion + sham groups. No differences were observed between the 6-OHDA-lesion group and the 6-OHDA-lesion + sham group (the data are listed in Supplementary Table 3). Thus, the treatment of PD rats with PNU-282987 significantly improved the 6-OHDA-induced Parkinsonian symptoms.

### PNU-282987 Attenuates the 6-OHDA-Mediated Dopaminergic Cell Loss in the SN

**FIGURE 3:**
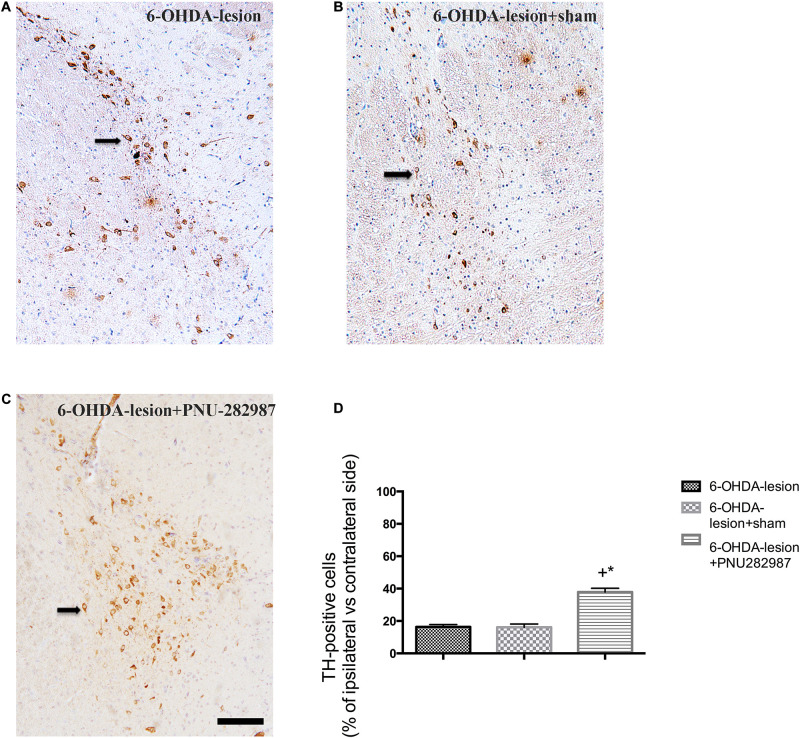
PNU-282987 attenuates the 6-OHDA-mediated loss of dopaminergic cells in the left side of SN. The images illustrate the SN immunostained with TH and a nuclear counterstain with hematoxylin staining. Scale bar indicates 200 μm. **(A)** 6-OHDA-lesion group; **(B)** 6-OHDA-lesion + sham group; **(C)** 6-OHDA-lesion + PNU-282987 group. **(D)** The percentages of TH-positive cells on the damaged side of the SN/cells on the contralateral side among three groups. ^+^*p* < 0.05 compared with the 6-OHDA-lesion group (*n* = 6 per group), and *p < 0.05 compared with the 6-OHDA-lesion + sham group (*n* = 6 per group).

The ability of PNU-282987 to protect against nigrostriatal dopamine neuron lesions induced by 6-OHDA was explored by performing TH immunostaining of the SN. According to our previous study (Jiang et al., 2018), the number of TH-positive cells on the damaged side of the SN was markedly decreased after 6-OHDA injection. However, as shown in Figure 3, the percentage of TH+ cells on the lesioned side with respect to the non-lesioned one was increased after PNU-282987 treatment (37.87 ± 2.86%) compared to the 6-OHDA-lesion (16.33 ± 1.46%) and 6-OHDA-lesion + sham groups (16 ± 2.1%) in our present study (One-way ANOVA, *F* = 38.922, *p* < 0.001). In addition, the following LSD *post hoc* test showed that the significant improvement of the 6-OHDA-lesion + PNU-282987 group with respect to the 6-OHDA-lesion group (*p* < 0.001) and 6-OHDA-lesion + sham group (*p* < 0.001). Based on these findings, PNU-282987 protected against the 6-OHDA-induced impairments in dopaminergic neurons.

### PNU-282987 Inhibits Neuroinflammation in the SN Induced by 6-OHDA Lesions

**FIGURE 4:**
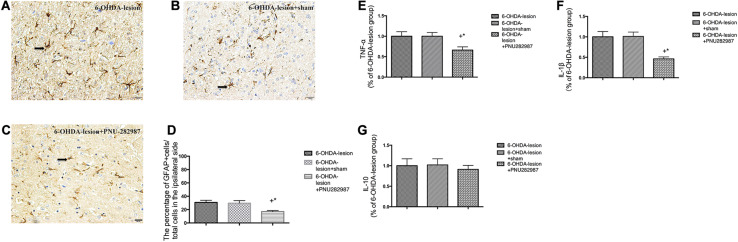
PNU-282987 exerted anti-neuroinflammation effects in 6-OHDA-lesioned rats. The morphological changes in activated GFAP cells in the SN were assessed using immunostaining and a nuclear counterstain with hematoxylin staining. Representative photomicrographs of the SN area are shown **(A–C)**. The scale bar indicates 20 μm. **(A)** 6-OHDA-lesion group; **(B)** 6-OHDA-lesion + sham group; **(C)** 6-OHDA-lesion + PNU-282987 group. **(D)** The graph depicts the percentage of GFAP+ cells in the SN/total cells on the lesioned side (counterstained with hematoxylin) for each experimental group. ^+^*p* < 0.05 compared with the 6-OHDA-lesion group (*n* = 6 per group), and *p < 0.05 compared with the 6-OHDA-lesion + sham group (*n* = 6 per group). **(E–G)** The mRNA levels of pro-inflammatory (TNF-α and IL-1β) and anti-inflammatory cytokines (IL-10) in the ventral midbrain region were analyzed using real time RT-PCR. The results were normalized to the 6-OHDA-lesion group. **(E)** TNF-α; **(F)** IL-1β; **(G)** IL-10; ^+^*p* < 0.05 compared with the 6-OHDA-lesion group (*n* = 6 per group), and *p < 0.05 compared with the 6-OHDA-lesion + sham group (*n* = 6 per group).

The mRNA levels of pro-inflammatory (TNF-α and IL-1β) and anti-inflammatory cytokines (IL-10) were determined to measure the extent of neuroinflammation (Figures 4E–G). In addition, the activated GFAP cells in the SN were also measured by performing immunohistochemistry (Figures 4A–D). As expected, the 6-OHDA-lesion + PNU-282987 group presented significantly downregulated mRNA levels of inflammatory cytokines [TNF-α (*p* = 0.032) and IL-1β (*p* = 0.002)] in the ventral midbrain compared with the 6-OHDA-lesion and 6-OHDA-lesion + sham groups, while no significant difference in the level of the anti-inflammatory cytokine (IL-10) was observed among three groups (One-way ANOVA followed by the LSD *post hoc* test). Moreover, the percentage of GFAP-positive cells was reduced after the PNU-282987 treatment (20.95 ± 1.53%) compared to the 6-OHDA-lesion (35.0 ± 2.33%) and 6-OHDA-lesion + sham groups (31.66 ± 1.75%) (One-way ANOVA; *F* = 14.94, *p* < 0.001). Also, the decrease in the 6-OHDA-lesion + PNU-282987 group was significant with respect to the 6-OHDA-lesion group (LSD *post hoc* test, *p* < 0.001) and 6-OHDA-lesion + sham group (LSD *post hoc* test, *p* = 0.001). Therefore, PNU-282987 has the ability to modulate the expression of inflammatory cytokines and suppress astrocyte overactivation under 6-OHDA-induced lesion conditions.

### Effect of PNU-282987 on Changes in CD4+ T Lymphocyte Subsets in the SN

**FIGURE 5:**
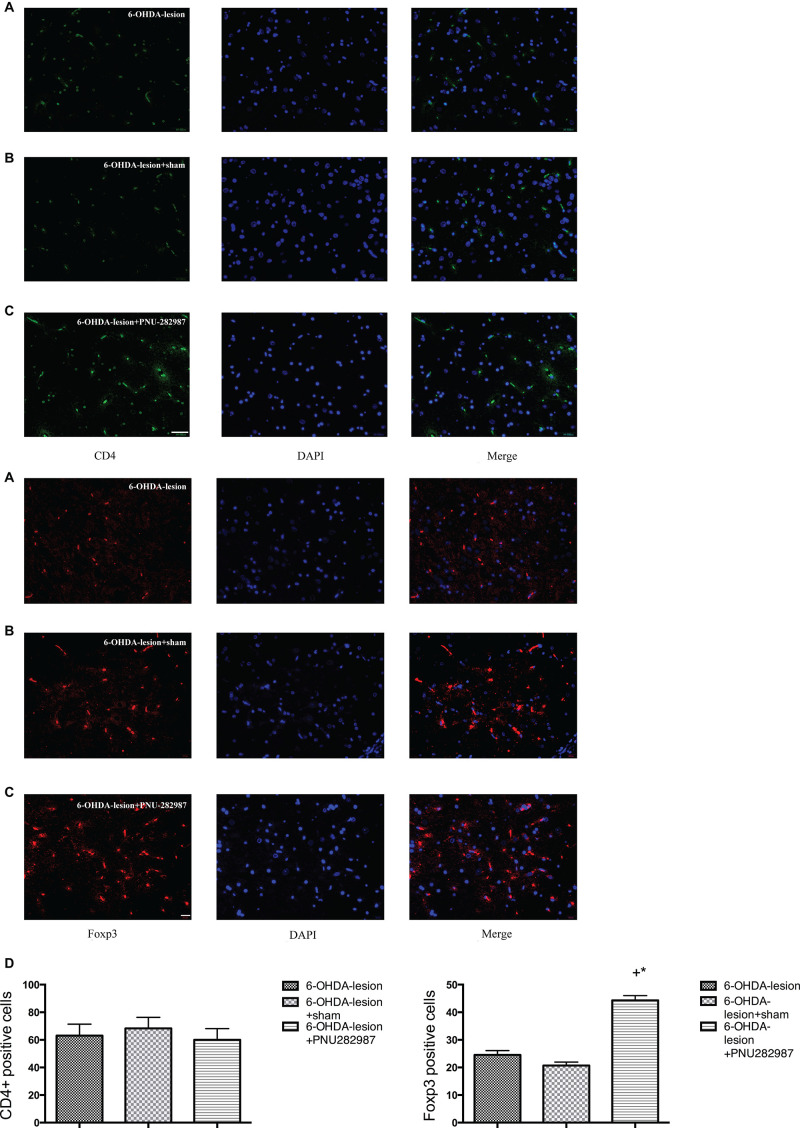
PNU-282987 changes the subsets of CD4+ T lymphocytes and Foxp3 cells in the SN. The immunofluorescence staining of Foxp3/DAPI was performed using antibodies against: CD4 (green stain) and Foxp3 (red stain) and DAPI (blue stain). Merged image showing CD4 or Foxp3 and DAPI staining (*n* = 6 per group). The scale bar indicates 200 and 20 μm, respectively. **(A)** 6-OHDA-lesion group; **(B)** 6-OHDA-lesion + sham group; **(C)** 6-OHDA-lesion + PNU-282987 group. **(D)** The graph depicts the positive number of CD4+ cells and Foxp3 cells in the SN from each experimental group. ^+^*p* < 0.05 compared with the 6-OHDA-lesion group (*n* = 6 per group), and *p < 0.05 compared with the 6-OHDA-lesion + sham group (n = 6 per group).

We examined the CD4+ T lymphocytes and the expression of Foxp3 in the SN of the rats belonging to the three groups by performing immunofluorescence staining to assess the effect of PNU282987 on immune responses in the PD rat model. Foxp3 is considered an important marker, as well as a key transcription factor in regulating the differentiation and function, of Treg cells (Mohr et al., 2019). As shown in Figure 5, CD4+ T lymphocytes infiltration was observed in the SN following the 6-OHDA injection. Moreover, the treatment of 6-OHDA-induced PD rats with PNU-282987 further increased the number of Foxp3 cells in the SN (44.33 ± 1.67) compared with the 6-OHDA-lesion (24.5 ± 1.61) and the 6-OHDA-lesion + sham groups (20.67 ± 1.28) (One-way ANOVA, *F* = 69.1, *p* < 0.001). In addition, the increase in the 6-OHDA-lesion + PNU-282987 group was significant with respect to the 6-OHDA-lesion group (LSD *post hoc* test, *p* < 0.001) and 6-OHDA-lesion + sham group (LSD *post hoc* test, *p* < 0.001). Based on these results, PNU-282987 mediated the CD4+ T cell differentiation process and the maintenance of the Treg population.

### Mechanisms Underlying the PNU-282987-Mediated Immune Balance in a PD Rat Model

**FIGURE 6:**
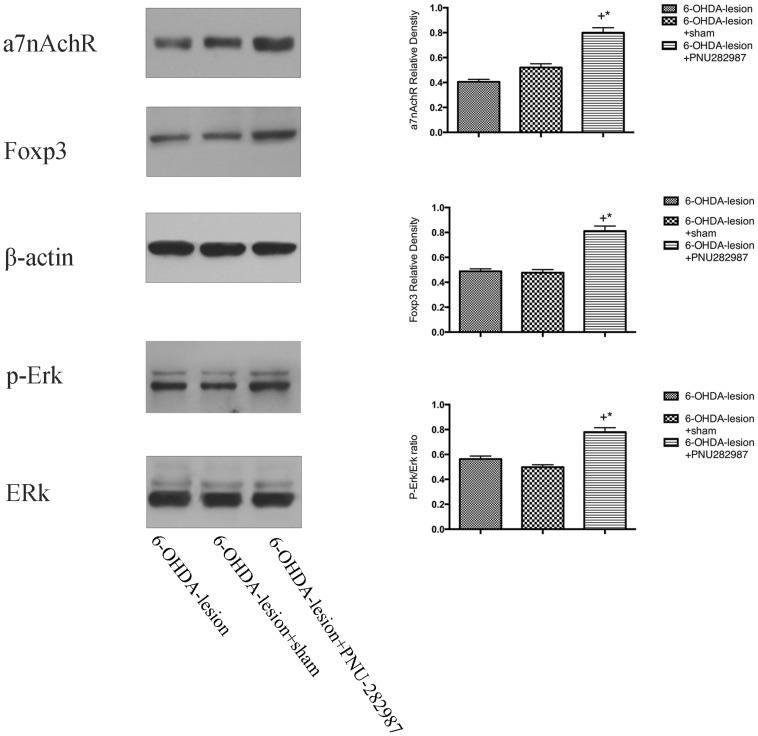
Mechanisms underlying the PNU-282987-mediated regulation of the immune balance in the PD rat model. The differences in the expression of the α7nAChRs, p-Erk/Erk and Foxp3 proteins among the three groups were determined using Western blotting. β-actin was used as a loading control to ensure equal protein loading. ^+^*p* < 0.05 compared with the 6-OHDA-lesion group (*n* = 6 per group), and *p < 0.05 compared with the 6-OHDA-lesion + sham group (*n* = 6 per group).

We detected the levels of α7nAChR, p-Erk/Erk and Foxp3 proteins in the ventral midbrain using Western blotting to understand the mechanisms by which PNU-282987 regulated immune responses in the PD rat model. As shown in Figure 6, compared with the 6-OHDA-lesion and 6-OHDA-lesion + sham groups, the 6-OHDA + PNU-282987 group exhibited increased levels of the α7nAChR and Foxp3 protein and activated Erk (expressed as the ratio of p-Erk/Erk) (One-way ANOVA, *p* < 0.05). In addition, no differences in the levels of these proteins were detected between the 6-OHDA-lesion and 6-OHDA-lesion + sham groups. Taken together, the PNU-282987 treatment increased the expression of Foxp3 possibly through the α7nAChR-mediated increase in p-Erk activity.

## Discussion

Neuroinflammation and the innate immune response have recently received increasing attention in the field of PD (Schonhoff et al., 2020; Siracusa et al., 2020). Interesting evidence has shown that PD is characterized by both altered levels of classic immune cells, such as microglia and astrocytes, and the infiltration of T cells, likely due to blood-brain barrier dysfunction (Wheeler et al., 2014). Moreover, neuroinflammation is thought to directly participate in the adaptive immune system (Bagheri et al., 2020). Autopsy studies of postmortem specimens from patients with PD and MPTP mouse models have demonstrated infiltration of CD4+ helper T cells into the nigrostriatum (González et al., 2013), suggesting that immune dysfunction might be a potential mechanism underlying the process of dopaminergic neurodegeneration in PD. Based on accumulating evidence obtained from recent studies, infectious agents, commensal bacteria and pathogenic forms of α-syn may trigger an innate immune response in the periphery and central nervous system, leading to neuroinflammation and dopaminergic cell death (Zhu et al., 2017; Comi et al., 2019; Pei and Maitta, 2019). Taken together, the results indicate that inflammatory and abnormal immune responses play crucial roles in PD. According to previous study (Truong et al., 2006), unilateral injection of a large dose of 6-OHDA (16 mg) into the MFB induced an complete injury PD model, while a low dose (4 mg) induced a partial injury. In our present study, we used a moderate dose (8 mg) to rapidly reproduce a late stage of PD with a unilateral injury.

Emerging evidence shows that α7nAChR activation reduces inflammatory responses in PD pathology (Quik et al., 2013; Stuckenholz et al., 2013; Foucault-Fruchard and Antier, 2017). In addition, the nicotinic acetylcholine receptors (nAChRs) mediate the process of murine splenic CD4+ T cell differentiation (Wang et al., 2010). In our previous studies, aVNS treatment suppressed the levels of inflammatory cytokines and increased α7nAChR expression in the ventral midbrain of 6-OHDA-treated rats (Jiang et al., 2018). Moreover, aVNS therapy improved PD-related immune disorders by altering the percentage of subsets of CD4+ T cells. Based on these findings, we concluded that the α7nAChR-mediated protection against nigrostriatal damage might be associated with anti-inflammatory processes and maintenance of the immune balance.

As shown in our previous study (Jiang et al., 2018), the 6-OHDA-treated rats required a prolonged amount of time to traverse the beam and exhibited a shorter latency to fall from the rotarod apparatus. Consistent with the behavioral impairments, the 6-OHDA treatment markedly decreased TH immunoreactivity in the SN. In the present study, we also adopted 6-OHDA as an exogenous toxin to initiate the PD neurodegenerative process. The results obtained in the present study reveal that the α7nAChR agonist PNU-282987 partially improved the motor behavior of 6-OHDA-lesioned rats and attenuated the loss of dopaminergic cells in the SN. Thus, the PNU-282987 treatment exerts a positive effect on 6-OHDA-induced insults.

According to accumulating evidences, 6-OHDA exacerbates the loss of dopaminergic neurons in part by inducing inflammatory mechanisms (Zhang et al., 2019; Zhuang et al., 2020). Some studies have reported enhanced immunoreactivity of GFAP in the striatum and SN of patients with PD and MPTP-induced mouse models (Brochard et al., 2009; Rostami et al., 2020). Consistent with these previous studies, our results revealed astrocyte activation in the SN following the 6-OHDA treatment. However, the PNU-282987 treatment suppressed the overactivation of astrocytes in the SN and the levels of inflammatory cytokines, suggesting that PNU-282987 might exert neuroprotection against 6-OHDA-induced injury partially through the modulation of the inflammatory response and the activity of immune cells.

In addition, we also observed significant infiltration of CD4+ T lymphocytes, particularly in the SN of 6-OHDA lesioned rats. CD4+ T cells might be involved in 6-OHDA-mediated pathology and affect motor behavioral deficits (Jiang et al., 2018). Our findings agree with previous studies (Brochard et al., 2009; Kustrimovic et al., 2016), suggesting that CD4+ T cell infiltration in the SN might be highly associated with dopaminergic cell loss as shown by immunofluorescence staining. We then further investigated the effect of α7nAChR activation on the differentiation of CD4+ T cells using Western blotting technology.

Tregs and Th17 cells constitute a distinct lineage of CD4+ T cells dedicated to maintaining the dynamic immune balance (Liu et al., 2017). Notably, Tregs play a critical role in the maintenance of immunological homeostasis and tolerance, as well as the suppression of effector T cell immune activation (Pahan and Pahan, 2019). The upregulation of Treg function or increases in the number of cells might be beneficial to the treatment of autoimmune diseases (Su et al., 2018). In addition, the current evidence on Treg differentiation and function is mainly driven by analyses of Foxp3 expression (Bacchetta et al., 2007). Foxp3 is an important marker and transcription factor required for the development and function of Tregs, which is important for maintaining the inner immune balance (Mohr et al., 2019). As shown in a previous study, mice expressing mutant Foxp3 exhibit impaired Treg cell activity, and this impairment is reversed by the transgenic expression of wild-type Foxp3 in these animals (Gavin et al., 2007). Interestingly, we found a greater increase in the expression of Foxp3 in the 6-OHDA + PNU-282987-treated group than in the 6-OHDA-lesion group, which revealed that the neuroprotective effect of α7nAChR activation might be partially due to the regulation of CD4+T cell differentiation, particularly Treg differentiation. Based on these findings, the activation of α7nAChRs might exert beneficial effects on PD not only by decreasing neuroinflammation but also by regulating the differentiation of the CD4+ T cell subsets, which might be a promising therapeutic approach for PD. However, the mechanisms by which α7nAChR modulates the T cell-mediated immune response in PD remain to be established.

The MEK/Erk pathway plays a crucial role in a wide variety of cellular functions, such as cell proliferation, cell cycle arrest, terminal differentiation and apoptosis (Cao et al., 2013). Activated Erk proteins translocate into the nucleus and activate transcription factors, and this activation regulates growth factor-induced gene regulation, cell cycle entry, or cell differentiation (Lavoie et al., 2020). Previous studies have shown that the Erk or JNK pathways are involved in adaptive Treg differentiation (Cheng et al., 2019; Wang et al., 2020). Hu et al. (2015) reported that B7C, a superior AChE inhibitor, promotes neurite outgrowth in PC12 cells by activating the α7nAChR/Erk pathway, whereas the blockade and genetic depletion of α7nAChR partially abolishes neurite outgrowth and Erk activation. AP-1 is one of the downstream targets of the MAPK signaling cascade and consists of four subfamilies: the Jun (c-Jun, JunB, and JunD), Fos, Maf and ATF-activating transcription factor protein families (Ogawa et al., 2014). These transcription factors play a central role in the immune system, including T cell activation, Th differentiation and exhaustion. Katagiri et al. (2019) recently found that JunBfl/fl Cd4- Cre mice exhibited a significant reduction in the number of Treg cells and that JunB–/– CD4+ T cells failed to differentiate into Treg cells *in vitro*, indicating that JunB plays a crucial role in the development of Tregs in a DSS-induced colitis model. Bao et al. (2016) also found that AP-1 regulated the activity of the Foxp3 promoter in Treg cells and that adenosine promoted Foxp3 expression in Treg cells during sepsis. In our study, we observed significantly higher levels of the α7nAChR, p-Erk and Foxp3 proteins in the 6-OHDA-lesion + PNU-282987 group than in the 6-OHDA-lesion group. Overall, these results suggested that the α7nAChR/p-Erk pathway contributes to the expression of Foxp3 and subsequently promotes the differentiation and function of Tregs, as well as the regulation of intracellular molecules related to effector T cells, possibly by increasing the expression of AP-1. Therefore, there is a further need to corroborate the effect of the a7nAchR/p-Erk pathway on the AP-1 expression.

## Conclusion

The activation of α7nAChR effectively exhibited anti-inflammatory activity and an immunoregulatory function in PD animal models. Additionally, the mechanism underlying the maintenance of the inner immune balance possibly involved the α7nAChR/p-Erk/Foxp3 signaling pathway. Further studies will focus on the role of a7AchR activation on the interactions among other effector T cells and investigate other possible mechanisms involved. Based on our findings and the data available in the literature, selective a7AchR agonists might represent a potential preclinical and clinical novel immunomodulating therapy.

## Data Availability Statement

The raw data supporting the conclusions of this article will be made available by the authors, without undue reservation, to any qualified researcher.

## Ethics Statement

All the experiments followed the Regulations of Experimental Animal Administration issued by the State Committee of Science and Technology of China.

## Author Contributions

YJ, LL, and TF conceived and designed the experiments. YJ, HM, XW, ZW, YY, and LL performed the experiments. YJ and TF analyzed the data. YJ and YY contributed reagents, materials, and analysis tools. YJ, LL, and TF contributed to the writing of the manuscript. All authors contributed to the article and approved the submitted version.

## Conflict of Interest

The authors declare that the research was conducted in the absence of any commercial or financial relationships that could be construed as a potential conflict of interest.

## Abbreviations

PD: Parkinson’s disease
α 7nAChR α 7: nicotinic acetylcholine receptors
6-OHDA: 6-hydroxydopamine
TH: tyrosine hydroxylase
SN: substantia nigra
Foxp3: forkhead/winged helix transcription factor p3
GFAP: glial fibrillary acidic protein
PCR: real-time polymerase chain reaction
Th: T helper
Fas: fibroblast-associated
Tregs: regulatory T cells
TGF- β 1: transforming growth factor
aVNS: auricular vagus nerve stimulation
MFB: medial forebrain bundle
PBS: phosphate-buffered saline
SDS-PAGE: SDS-polyacrylamide gel electrophoresis
SEM: standard error of the mean
ANOVA: one-way analysis of variance
LSD: least significant difference
HRP: horseradish peroxidase
p-Erk: phosphorylated extracellular signal-regulated kinase
MEK/Erk: mitogen-activated protein/extracellular signal-regulated kinase
JNK: Jun N-terminal kinase
AP-1: activator protein-1.
