# Supplementary material for: Protective Effect of the α7 Nicotinic Receptor Agonist PNU-282987 on Dopaminergic Neurons Against 6-Hydroxydopamine, Regulating Anti-neuroinflammatory and the Immune Balance Pathways in Rat
Source: Front Aging Neurosci. 2021 Jan 25;12:606927. doi: 10.3389/fnagi.2020.606927 (PMC7868536; doi:10.3389/fnagi.2020.606927)
Supplement: Supplementary file 1 [file Data_Sheet_1.PDF]

Table 1 The values of apomorphine-induced rotation in three groups (Turns/h)

Behavior test (Turns/h)	6-OHDA-lesion group	6-OHDA-lesion +sham group	6-OHDA-lesion +PNU-282987 group
Apomorphine-induced rotational behavior	632	574	378
	517	627	413
	577	494	536
	543	557	519
	584	509	452
	513	593	470

Table 2 The values of Rotarod test in three groups (s)

group/ Rotarod test(s)	Trial 1	Trial 2	Trial 3
6-OHDA-lesion group	40.12	38.79	42.43
	27.66	30.13	26.45
	33.62	28.99	35.71
	17.00	19.93	20.45
	32.45	37.83	39.67
	22.77	20.48	28.95
6-OHDA-lesion +sham group	34.78	29.65	35.67
	16.33	21.62	20.99
	25.79	32.67	30.12
	44.78	47.49	40.32
	28.53	22.79	25.80
	22.23	19.19	15.56
6-OHDA-lesion +PNU-282987 group	132.78	156.74	149.35
	90.17	82.65	76.79
	76.53	81.47	88.57
	154.75	138.9	142.79
	132.46	127.07	128.94
	116.55	135.47	142.25

Table 3 The values of Beam-walking test in three groups (s)

group/ Beam-walking test(s)	Trial 1	Trial 2	Trial 3
6-OHDA-lesion group	20.83	30.40	21.41
	36.99	34.68	36.94
	37.55	37.99	39.72
	27.77	24.25	20.19
	37.90	35.01	30.33
	34.69	40.61	36.71
6-OHDA-lesion +sham group	29.24	24.65	27.62
	43.95	39.38	43.85
	32.48	28.95	40.56
	21.45	18.03	29.70
	31.07	29.63	28.46
	40.84	36.61	25.14
6-OHDA-lesion +PNU-282987 group	12.65	10.89	9.59
	26.35	22.32	22.43
	27.71	32.73	26.60
	16.95	15.83	13.42
	21.05	20.51	19.76
	27.13	30.09	29.18
